# Intratumoral expression of CCR3 in breast cancer is associated with improved relapse-free survival in luminal-like disease

**DOI:** 10.18632/oncotarget.8680

**Published:** 2016-04-11

**Authors:** Di-He Gong, Lei Fan, Hai-Yan Chen, Ke-Feng Ding, Ke-Da Yu

**Affiliations:** ^1^ Department of Breast Surgery, Shanghai Cancer Center and Cancer Institute, Shanghai Medical College, Fudan University, Shanghai, 200032, P. R. China; ^2^ Department of Thyroid and Breast Surgery, Affiliated Cixi Hospital, Wenzhou Medical University, Cixi Zhejiang, 315300, P. R. China; ^3^ Department of Surgical Oncology, The Second Affiliated Hospital of Zhejiang University School of Medicine, Hangzhou Zhejiang, 310009, P. R. China

**Keywords:** CCR3, breast cancer, peritumoral, luminal-like, relapse-free survival

## Abstract

**Purpose:**

The association chemokine receptor CCR3 with breast cancer subtypes and relapse-free survival is unknown.

**Results:**

The overall expression (either intratumoral or peritumoral) of CCR3 was not associated with tumor size, lymph node status, age, and subtype. When we confined the analysis in samples without peritumoral stromal CCR3 expression, intratumoral expression of CCR3 was associated with breast cancer subtype (P=0.04). Tumors with high expression of CCR3 were more likely to be luminal-like rather than TNBC or HER2-enriched cancers. Moreover, high mRNA expression of CCR3 was related with improved relapse-free survival in luminal-A/B (P<0.001). The subsequent sensitivity analysis using the systemically untreated patients confirmed that higher mRNA expression of CCR3 was a robust prognostic factor for luminal-A (P=0.0025) and luminal-B (P=0.088), but not for HER2-enriched (P=0.21) and TNBC (P=0.86). In the independent cohort, the positive association between increased expression of CCR3 and improved distant relapse-free survival was also observed.

**Methods:**

We determined the expression level of CCR3 in 150 cases with breast cancer by using immunohistochemistry (IHC) assay, for both intratumoral and peritumoral stroma, and investigated the effect of CCR3 expression on relapse-free survival according to subtype using cases from publicly available datasets, in the whole group (N=3557) and in the patients without adjuvant systemic treatment (N=1005), respectively. Moreover, the survival outcomes were validated in another independent cohort including 508 breast cancer patients treated with neoadjuvant chemotherapy.

**Conclusions:**

Our data indicate that intratumoral expression of CCR3 in breast cancer is associated with improved relapse-free survival in patients with luminal-like disease.

## INTRODUCTION

Recent development of gene expression microarray technology has made the intrinsic molecular classification of breast cancer possible [1]. Different subtypes of breast cancer exhibit diverse behaviors, varied prognosis, as well as different response to systemic treatment [2]. Among the known four subtypes (luminal-A, luminal-B, HER2-enriched, and triple-negative breast cancer [TNBC]), TNBC and HER2-enriched subtypes display more aggressive behaviors compared with luminal-like disease, which is defined by the moderate to adequate expression of estrogen receptor (ER) and/or progesterone receptor (PR).

Cytokines are a protein family of regulatory factors derived from tumors and their environmental components that contribute to the growth, invasion, and metastasis of cancer. CCR3 is identified to be one of the major factors that involve in the progression and metastasis of some human tumors [3–5]. For instance, CCR3/eotaxin-1 loop has been revealed to increase the growth of malignant tumor cells in T-cell lymphomas [4], and the presence of CCR3 in human renal cell carcinoma samples correlates with the grade of malignancy [5]. Moreover, CCR3 and CCR10 are the known receptors for CCL28 [6], which could promote tumor tolerance and angiogenesis [7]. However, to our best knowledge, few studies have addressed the association between CCR3 and breast cancer, especially for different breast cancer subtypes. Interestingly, a previous report regarding the analysis of the expression profiles of cytokines and cytokine-related genes during the progression of breast cancer growth in mice showed that CCR3 was up-regulated in tumor tissues, but down-regulated in lymph nodes during tumor growth [8], indicating that CCR3 might be a protective factor during cancer development and metastasis.

The aim of this study was to detect the expression of CCR3 in both breast tumor and tumor stroma, and to investigate the association of CCR3 expression with different breast cancer subtypes. Furthermore, the effect of CCR3 on survival was explored in publicly available databases.

## PATIENTS AND METHODS

### Study patients and samples

The tumor samples used in this study were from patients with operable primary invasive breast carcinoma (stages I to IIb and T3N1M0) who received surgical therapy between May-2010 and March-2013, at the Department of Thyroid and Breast Surgery in Affiliated Cixi Hospital of Wenzhou Medical University. Tumors were staged according to the American Joint Committee on Cancer (AJCC) pathologic tumor-node-metastasis (TNM) classification (the 7^th^ edition). One hundred and fifty consecutive cases were included in this study and the basic information of clinical and pathological characteristics are shown in Table 1. Exclusion criteria included neoadjuvant chemotherapy, prior malignancies, and stage IV disease. All patients were female, with the median age of 52 years (ranging from 24 to 75 years). The research protocol was reviewed and approved by the Ethical Committee of the Affiliated Cixi Hospital of Wenzhou Medical University. All participants provided written informed consents.

**Table 1:** Basic information of clinical and pathological characteristics of 150 patients with breast cancer

		Number	%
Median age (min to max), years		52 (24 to 85)	
Tumor size	≤2 cm	17	11.3
	>2 cm	133	88.7
Lymph node	Negative	93	62.0
	Positive	57	38.0
ER status	Negative	53	35.3
	Positive	97	64.7
PR status	Negative	56	37.3
	Positive	94	62.7
HER2 status	Negative	113	75.3
	Positive	37	24.7
Subtype	Luminal-A	86	57.3
	Luminal-B (HER2-positive)	21	14.0
	Triple-negative	27	18.0
	HER2-enriched	16	10.7
CCR3 (intratumoral expression)	Negative	20	13.3
	Positive	130	86.7
CCR3 (peritumoral stromal expression)	Negative	129	86.0
	Positive	21	14.0

### Measurement of ER, PR, and HER2 status

Immunohistochemistry (IHC) assessment of ER, PR, and HER2 expression was conducted in paraffin-embedded tumor samples obtained from surgical operation according to American Society of Clinical Oncology/College of American Pathologists (ASCO/CAP) guidelines [9, 10]. ER and PR are considered positive if there are at least 1% positive tumor nuclei in the sample on testing in the presence of expected reactivity of internal and external controls [9]. HER2 positivity was determined by IHC 3+ (HerceptTest, DAKO, Denmark) or fluorescence in situ hybridization (FISH) positive status (PathVysion HER2 DNA probe kit) [10]. Molecular subtype was categorized as following [11, 12]: luminal-A, ER and/or PR positive and HER2 negative; luminal-B, ER and/or PR positive and HER2 positive; HER2-enriched, ER and PR negative and HER2 positive; TNBC, ER, PR, and HER2 negative. Consequently, there were 86 (57%) luminal-A, 21 (14%) luminal-B, 27 (18%) TNBC, and 16 (11%) HER2-enriched breast cancers.

### IHC assay for CCR3

IHC assay of CCR3 expression was performed, for both intratumoral and peritumoral stroma. The procedure of IHC assay had been described previously [13]. In brief, four-micron paraffin sections were prepared, and tissue sections were deparaffinized in xylene for 5 minutes and rehydrated with graded ethanol. The endogenous peroxidase activity was blocked with 3% hydrogen peroxide for 10 minutes. Antigen retrieval was done with 10 mM citrate buffer. The sections were incubated with diluted goat serum for 10 minutes followed by incubated overnight with the primary CCR3 rabbit monoclonal antibody (dilution 1:500, Rabbit monoclonal to CCR3, clone ab32512, Abcam Corp. Cambridge, UK) at 4°C. The slides were incubated with biotinylated secondary antibody for 30 minutes and then the slides were incubated for 30 minutes with streptavidine-peroxidase. Staining development was performed with 3-3′-diaminobenzidine. Negative controls were carried out by replacement of the primary antibody with substituting phosphate buffer saline.

**Figure 1:**
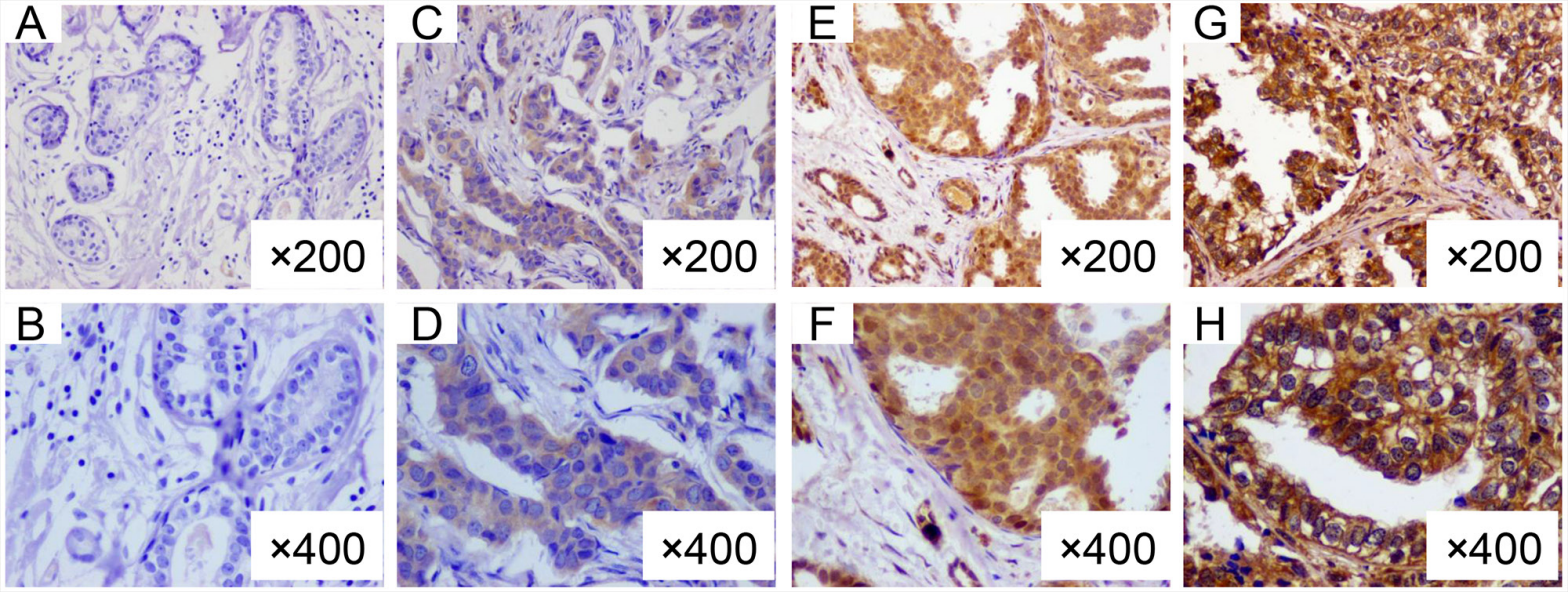
Representative immunohistochemical staining of CCR3 in breast cancers **A.** (×200) and **B.** (×400), intratumor negative and peritumoral stroma negative for CCR3; **C.** (×200) and **D.** (×400), intratumor moderate positive and peritumoral stroma negative; **E.** (×200) and **F.** (×400), intratumor strong positive and peritumoral stroma negative; **G.** (×200) and **H.** (×400), intratumor positive and peritumoral stroma positive for CCR3

The immunereactivity was evaluated according to Hao's method [14]. Briefly, immunostaining was independently examined by two clinical pathologists who were unaware of the patient's clinico-pathologic information. For each sample, five high-power fields (200×) were randomly selected. Cytoplasmic and membranaceous staining intensity and percentage of positive tumor cells were assessed. The extent of staining was categorized into five semi-quantitative classes based on the percentages of positive tumor cells: 0 (<5% positive cells), 1 (6 to 25% positive cells), 2 (26 to 50% positive cells), 3 (51 to 75% positive cells), and 4 (>75% positive cells). The intensity of staining was also determined semi-quantitatively on a scale of 0 to 3 as followings: 0 (negative), 1 (weak staining), 2 (moderate staining), and 3 (strong staining). Multiplication of the intensity and the percentage scores gave the final staining scores [14]: 0 to 2, as negative; 3 to 12, as positive. Disparity of results between the two pathologists were resolved by discussion, and a consensus was reached. The representative CCR3 IHC staining plots are shown in Figure 1.

### Survival analysis

Because of short follow-up time and rare event among our study cases, we could not analyze survival outcome in our cohort. Instead, we conducted the survival analysis in an online tool using the publicly available datasets. This online database named Kaplan Meier plotter was established using gene expression data and survival information of 4142 breast cancer patients (2014 version) downloaded from Gene Expression Omnibus (GEO). CCR3 gene (probe set, 208304_at) was entered into the database (http://kmplot.com/breast/, last accessing time 2015-12-20) to obtain Kaplan-Meier survival plot where the number-at-risk was indicated below the main plot [15, 16]. Hazard ratio (HR) with 95% confidence intervals (CI) and log-rank P were calculated and displayed on the webpage. The primary endpoint of interest was relapse-free survival (RFS). We investigated the effect of CCR3 expression on RFS by breast cancer subtype, both in all the patients (N=3557) and in the patients without adjuvant systemic treatment (N=1005, received neither adjuvant chemotherapy nor endocrine therapy).

### Validation cohort for survival outcome

To validate the association of CCR3 expression with survival, we chose an independent cohort of patients treated with neoadjuvant chemotherapy. This cohort from M.D. Anderson Cancer Center (MDACC, Houston, TX) has been described previously [17, 18]. Briefly, these 508 patients were those with newly diagnosed HER2-negative breast cancer treated with neoadjuvant chemotherapy containing sequential anthracycline and taxane-based regimens (then underwent surgery and followed by endocrine therapy if ER-positive). All gene expression microarrays were profiled in the Department of Pathology at the MDACC and the details of the methods for RNA purification and microarray hybridization have been reported previously [19, 20]. The study endpoint was distant relapse-free survival (DRFS), which was calculated from initial diagnostic biopsy of breast cancer to the occurrence of distant metastasis or non-breast cancer death. Data sets for this study are accessible via the GEO repository under accession identification numbers GSE25066 [18].

### Statistics

The association between CCR3 expression level and clinico-pathologic variables was assessed by χ2 test. The Kaplan-Meier method was used to draw survival plot. Two-sided P<0.05 was considered statistically significant. All of the statistical analysis was performed using SPSS 17.0 (SPSS Inc, Chicago, IL, USA).

## RESULTS

### Association between CCR3 expression and clinico-pathologic variables

We first evaluated the association between IHC expression level of CCR3 and clinico-pathologic characteristics in our study cohort. This analysis included 150 patients. However, we found that overall CCR3 expression (either intratumoral or peritumoral) was not associated with tumor size, lymph node status, age, and subtype (defined by ER, PR, and HER2 status)(data not shown).

### Intratumoral and peritumoral stromal expression of CCR3 in breast cancer by subtype

We noticed that, in a minority of cases (14%, 21/150), peritumoral stroma displayed positive CCR3. The interaction between intratumoral and peritumoral stromal expression of CCR3 is shown in Table 2. When the peritumoral stroma was CCR3 positive, the intratumoral expression of CCR3 would be positive; however, when intratumoral expression of CCR3 was positive, only a few cases would peritumorally positive.

**Table 2:** Association between intratumoral and peritumoral stromal expression of CCR3 (N=150)

		Peritumoral stromal expression of CCR3				P
		Negative	%	Positive	%	
Intratumoral expression of CCR3	Negative	20	15.5	0	0	0.05^*^
	Positive	109	84.5	21	100	

* Fisher exact test P=0.07

Since peritumorally expressed CCR3 might be a confounding factor for tumor initiation and subtype development, we therefore confined our analysis in samples without peritumoral stromal expression of CCR3. Interestingly, when peritumoral stromal CCR3 was weak or absent, intratumoral expression of CCR3 was associated with breast cancer subtype (P=0.04) (Table 3). Tumors with intratumoral CCR3 expression were more likely to be luminal-like rather than TNBC or HER2-enriched. Of note, HER2-enriched cases were more likely to be intratumoral CCR3 negative.

**Table 3:** Association of intratumoral expression of CCR3 with tumor size, node status, and subtype, in cases without CCR3 expression in peritumoral stroma (N=129)

		Intratumoral CCR3 negative		Intratumoral CCR3 positive		P
		Number	%	Number	%	
Tumor size	≤2 cm	3	20.0	12	80.0	0.61
	>2 cm	17	14.9	97	85.1	
Lymph node	Negative	10	12.7	69	87.3	0.26
	Positive	10	20.0	40	80.0	
Subtype	Luminal-A	10	13.7	63	86.3	0.04
	Luminal-B (HER2-positive)	1	5.3	18	94.7	
	Triple-negative	4	16.0	21	84.0	
	HER2-enriched	5	41.7	7	58.3	

### Survival analysis of CCR3 according to breast cancer subtype

**Figure 2:**
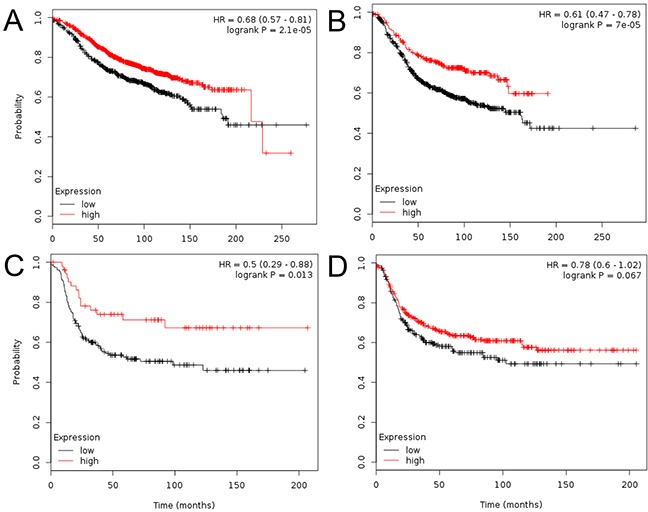
Kaplan-Meier plots derived from http://kmplot.com/analysis/ The analyses were performed and the Kaplan-Meier plots were drawn regarding CCR3 expression and relapse-free survival in the luminal-A **A.** luminal-B **B.** triple-negative **C.** and HER2-enriched **D.** The “best cutoff” for CCR3 mRNA was used.

**Figure 3:**
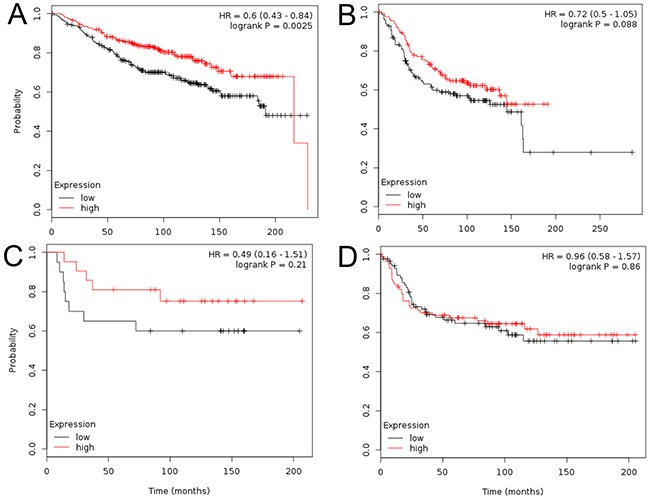
Kaplan-Meier plots in systemically untreated patients The patients were split by median expression of CCR3 mRNA. Plots are shown according to subtype: luminal-A **A.** luminal-B **B.** triple-negative **C.** and HER2-enriched **D.**

Subsequently, to evaluate the prognostic effect of CCR3, we constructed Kaplan-Meier plots and log-rank analyses in 3557 patients in an online database. At first, we performed this analysis using the “best cutoff” value of mRNA of CCR3 (all percentiles are computed and the best performing threshold is automatically chosen as the cutoff). In the whole population, high CCR3 expression was an indicator of reduced risk for relapse (P<0.001). When we analyzed according to subtype, the high expression of CCR3 was related to improved RFS in luminal-A (P<0.001, Figure 2A), luminal-B (P<0.001, Figure 2B), HER2-enriched (P=0.013, Figure 2C) cases, but not in TNBC (P=0.07, Figure 2D) cases. To confirm these findings, we performed a sensitivity analysis using the systemically untreated patients and using the median value of CCR3 mRNA as the cutoff. It showed that relatively higher expression of CCR3 was a robust prognostic factor for luminal-A (P=0.0025, Figure 3A) and luminal-B (borderline P=0.088, Figure 3B), but not for HER2-enriched (P=0.21, Figure 3C) and TNBC (P=0.86, Figure 3D).

### Validation for survival outcome

**Figure 4:**
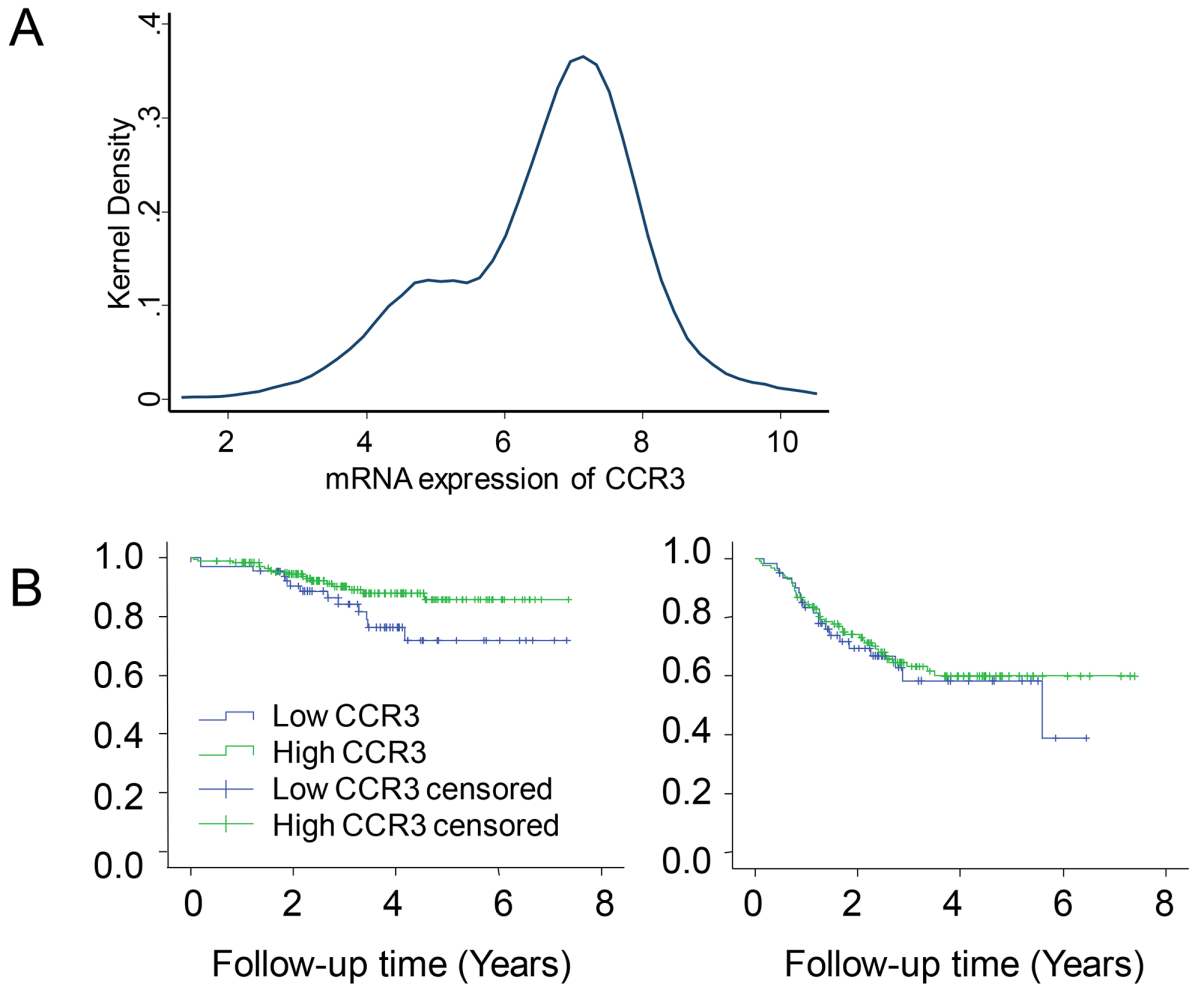
Validating the effect of CCR3 expression on distant relapse-free survival (DRFS) in patients receiving neoadjuvant chemotherapy The Kernel density estimate was used to determine the optimal cutoff of CCR3 mRNA level **A.** Kaplan-Meier plots are shown according to subtype: luminal-like **B, left,** and triple-negative **B, right**.

We chose the M.D. Anderson cohort for validation. The Kernel density estimate method was used to determine the optimal cutoff of CCR3 mRNA level (Figure 4A). Using the expression of 6.0 as the cutoff value, we analyzed DRFS according to PAM50-predicted subtypes. The higher expression of CCR3 was related to improved DRFS in luminal-like disease (P=0.068, Figure 4B left), but not in TNBC (P=0.62, Figure 4B right) or normal-like disease (P=0.82, plot not shown).

## DISCUSSION

In the present study, we found that the intratumoral expression of CCR3 was associated with luminal-like disease. Moreover, we demonstrated that higher intratumoral mRNA expression of CCR3 reduced the risk for cancer relapse in luminal-like disease but not in TNBC and HER2-enriched cancers.

Recent findings have suggested chemokines and their receptors play important roles in cancer biology by influencing the tumor microenvironment and regulating cancer cell function [21]. CCR3 is a novel receptor associated with development, progression, and aggressiveness of several types of cancer. In the present study, we examined its expression in luminal-A/B, TNBC, and HER2-enriched subtypes, and showed that the expression of CCR3 is universal (87% intratumoral expression). Of note, high CCR3 expression was more prevalent in luminal-like cases, indicating CCR3 might be involved in luminal-like cancer development. In contrast, CCR3 positive rate was relatively low in TNBC or HER2-enriched cancers, implied that the diverse effect of CCR3 in development of different breast cancer subtypes. Previous research also suggested that CCL5/CCR3 signaling pathway is involved in prognosis and immunotherapy of luminal breast cancer [22]. To the best of our knowledge, the current study represents the first comprehensive investigation reporting the positive association between CCR3 expression and luminal-like breast cancer. It warrants further investigation regarding the precise mechanism of CCR3 modulating luminal-like cancer.

Furthermore, higher expression of CCR3 is significantly associated with improved RFS in luminal-A and luminal-B cases, suggesting that CCR3 may reduce or delay metastases in breast cancer. We not only found the protective effect of CCR3 in unselected population, but also successfully repeated the outcomes in patients without adjuvant systemic treatment, indicating a robust and steady effect of CCR3 on breast cancer relapse. To validate the findings, we chose an independent cohort of 508 patients treated with neoadjuvant chemotherapy from MDACC. Again, higher expression of CCR3 was related to improved DRFS in luminal-like disease but not in TNBC or normal-like disease. Our study had some limitation. First, the association between intratumoral expression of CCR3 and luminal-like disease is only observed in our cohort of 150 cases, and needs further validation. Second, we measured the protein (by IHC) of intratumoral and peritumoral stromal expression of CCR3 in subtype association analysis while investigated the mRNA of CCR3 in survival analysis. There might be inconsistent between protein and mRNA. The last but not the least, despite of the large number of sample size and successful validation in the survival analysis, we must point out that the publicly available gene microarray databases from different institutions and laboratories could inevitably introduce bias.

In conclusion, we have shown for the first time that the higher mRNA expression of CCR3 indicates a decreased risk of relapse in luminal-A and luminal-B cases, but not in TNBC and HER2-enriched patients. The precise mechanism underlying the association between CCR3 and luminal-like disease deserves further investigation.
